# Physical and biological roles of mesoscale eddies in Japanese eel larvae dispersal in the western North Pacific Ocean

**DOI:** 10.1038/s41598-018-23392-5

**Published:** 2018-03-22

**Authors:** Yu-Lin K. Chang, Yasumasa Miyazawa, Mélanie Béguer-Pon, Yu-San Han, Kyoko Ohashi, Jinyu Sheng

**Affiliations:** 1 Application Laboratory, Japan Agency for Marine-Earth Science and Technology, Yokohama, 236-0001 Japan; 2 Department of Oceanography, Dalhousie University, Halifax, Nova Scotia B3H 4R2 Canada; 3 Institute of Fisheries Science, National Taiwan University, Taipei, 106 Taiwan

## Abstract

The physical and biological roles of mesoscale eddies in Japanese eel larvae dispersal are investigated using a three-dimensional (3D) particle-tracking method, with a focus on the Subtropical Counter Current eddies of the western North Pacific Ocean. Virtual eel larvae (v-larvae) movements depends on the 3D ocean currents and active swimming behavior, including vertical swimming (diel vertical migration), horizontal directional swimming toward settlement habitat, and horizontal swimming toward available food. V-larvae are able to remain in eddies passively due to mesoscale eddy nonlinearity and/or actively due to attraction to rich food supplies. Thus, both physical trapping and biological attraction to food contribute to the retention of v-larvae in eddies. Physical trapping dominates the retention of v-larvae whose swimming speeds are slower than the eddy propagation speed, whereas biological food attraction prevails in the retention of v-larvae swimming faster than eddy propagation. Food availability differs between warm (anti-cyclonic) and cold (cyclonic) eddies, with the latter providing a richer food supply. Fish larvae that are retained for longer durations in cold eddies (shorter durations in warm eddies) are able to obtain more food and potentially grow faster, which enhances survival rates.

## Introduction

Mesoscale eddies play an important role in various biochemical and physical processes^1,4^. The impact of eddies on biogeochemistry and marine life has been documented for decades^5–9^. Eddy-induced nutrient flux is considered to be an important constituent, contributing 20% to 50% to the global primary production^6,7^. It has been suggested that the abundant biological productivity in mesoscale eddies makes them favorable habitats for marine organisms^9–11^. High concentrations of chlorophyll, biomass, and production have been observed in cold eddies^12–14^. Previous numerical simulations also suggest that biological production is higher in cold (cyclonic) eddies than in warm (anti-cyclonic) eddies^15,16^. Migratory fish display affinities for fronts and eddies^10^ and high abundances of fish larvae have been observed in cold eddies, particularly near the margins^11^. Despite low productivity in warm eddies, fish larvae have also been observed there^5^, and adult migratory fish such as tuna have shown circular trajectories for up to 20 days in these habitats^10^. Changes in ocean productivity have been found to influence larvae feeding success and survival, particularly during their early life stages^17,18^. A recent study suggests that the rich nutrient supply in mesoscale eddies can lead to faster growth rates of larval coral reef fish and result in greater survival and settlement^19^.

In addition to their biochemical roles, mesoscale eddies also play a crucial physical function by transporting ocean mass. The 30–40 Sv (1 Sv = 10^6^ m^3^ s^−1^) of mass transported by global mesoscale eddies is comparable to that of large-scale wind- and thermohaline-driven circulations^2^. All mesoscale eddies outside the tropical region have been found to be nonlinear, which implies trapping of water masses within the eddy interior^1,20^. Therefore, mesoscale eddies may significantly influence the dispersal of slow-moving ocean organisms such as fish larvae and other zooplankton species. Eddies have been reported to transport nutrients and zooplankton from the Chukchi Sea to the Canada Basin in the Arctic Ocean, covering a distance of more than 150 km^21,22^. A recent numerical study suggests that trapping by a mesoscale eddy can accelerate or decelerate the dispersal of fish larvae (depending on the swimming speed of the organisms relative to the eddy propagation speed)^23^. Although the observed presence of fish larvae in mesoscale eddies can be attributed to passive physical trapping and/or active biological behavior, the relative importance of each mechanism remains to be studied.

Mesoscale eddies, with radii of O (100 km) and extending to depths of 200–1000 m, have a mean lifetime of 32 weeks and a mean propagation distance of 550 km^1–3^. These eddies are either cyclonic or anti-cyclonic and are frequently observed in the world’s oceans. Mesoscale eddies have mean rotation speeds of 0.1–0.2 m s^−1^ and propagate westward at baroclinic Rossby wave phase speeds of O (10^−2^ m s^−1^). The Subtropical Counter Current (STCC), a weak (~0.02 m s^−1^) eastward flow that extends from 130°E to 170°E and from 17°N to 27°N, is one of the most energetic eddy zones in the western North Pacific Ocean and comprises many mesoscale eddies with diameters of 150–300 km^1,24,25^. These mesoscale eddies are nonlinear and thus have the potential to trap fish larvae within them^3,26^.

The Japanese eel, *Anguilla japonica*, is a species of high economic importance but has been listed as endangered on the IUCN red list^27^. The Japanese eel’s spawning ground is located to the west of the Mariana Islands^28^, and after hatching, eel larvae drift for 5–6 months toward their recruitment areas, which are estuaries in East Asia^29^. Japanese eel larvae (leptocephali and meta-larvae) have been observed in the STCC eddy zone during autumn and early winter^29^. A recent numerical study also indicated that the STCC eddy area is part of the path of Japanese eel larvae’s shoreward migration to their East Asian settlement habitat^30^. This study aims to investigate, for the first time, the contributions of passive physical trapping and active biological food attraction to fish larvae distribution in mesoscale eddies using numerical results from both idealized and realistic experiments. The simulations in this study will focus on the dispersal of Japanese eel larvae in STCC eddies.

## Data and Methods

A three-dimensional (3D) particle-tracking method was used to examine the movement of virtual eel larvae (v-larvae). In the simulations, v-larvae were carried by ocean currents in addition to their own swimming behavior, as described in the following sections.

### Idealized experiments using the coupled bio-physical model

The coupled bio-physical model used in the idealized experiments comprised ocean circulation and biogeochemical components^31^. The former was based on the Message Passing Interface version of the Princeton Ocean Model (mpiPOM)^32,33^, and the latter^34^ received 3D currents and parameterized turbulence fields generated by mpiPOM. The biogeochemical processes specified in the model included phytoplankton growth, mortality, and aggregation; zooplankton grazing and mortality; and detritus remineralization and sinking. Both models have been validated against observational data^35–38^.

To examine the effects of mesoscale eddies, several idealized experiments were conducted using the coupled bio-physical model. The model had a horizontal resolution of 0.1° × 0.1° with 57 terrain-following vertical levels and its domain covered the STCC region between 118°E and 142°E and between 16°N and 30°N. The water depth in these experiments was spatially uniform at 1000 m and salinity was set to a constant value of 35 psu. The initial temperature and biological variables were horizontally uniform and vertically variable, based on domain-averaged STCC climatological profiles obtained from the World Ocean Atlas (http://www.nodc.noaa.gov/OC5/WOA05/pr_woa05.html). Solar radiation was fixed at 150 W/m^2^, based on the National Center for Environmental Prediction (NCEP) climatological net shortwave radiation in the western Pacific Ocean. For simplicity, all other external forcings in these idealized experiments were set to zero.

An isolated mesoscale eddy, with characteristics similar to those of observed STCC eddies, was injected into the model using the method suggested by Shaw^39^, which was recently applied to examine the effect of eddies on shelf currents^40,41^. The injected warm (anti-cyclonic) or cold (cyclonic) eddy had a diameter of 250 km and was centered at 135°E and 23°N. During a period of 10 days, the eddy was gradually ramped up through changes to surface heating or cooling within the eddy. During this spin-up period, an eddy velocity field formed in accordance with changes in the distribution of isopycnals. To conserve heat, a uniform upward or downward surface heat flux was specified over the entire model domain. Although the 10-day spin-up period was chosen based on previous studies^40,41^, the actual eddy growth time varies with environmental conditions. Following eddy spin-up, the model was integrated for 200 days.

### Realistic experiments using JCOPE2

The Japan Coastal Ocean Predictability Experiment 2 (JCOPE2), a reanalysis dataset produced using a data-assimilative ocean circulation model, was used in the realistic experiments. The JCOPE2 model was constructed from the Princeton Ocean Model using a generalized coordinate system^42^ and the model domain encompassed the western North Pacific (10.5°N–62°N and 108°E–180°E), with a horizontal resolution of 1/12° (8–9 km) and 46 vertical layers. The external forcings that drive JCOPE2 include wind stress and net fluxes of heat and freshwater at the sea surface. Satellite and *in-situ* temperature and salinity data were assimilated into the model using a 3D variational method^42^. Previous comparisons of simulated passive particle trajectories with observed data indicated satisfactory performance of JCOPE2 in simulating 3D circulation and hydrography over the western North Pacific Ocean^30^.

### Particle-tracking scheme

The 3D particle-tracking scheme, developed by Ohashi and Sheng^43^ based on the fourth-order Runge–Kutta method^44^, was used in this study. An identical tracking scheme with a time step of three hours was used previously by Chang *et al*.^23,30,45^ to investigate the dispersal of Japanese eel larvae in the western Pacific Ocean and was also used to simulate adult American eel migration in the Atlantic Ocean^46,47^.

Three Japanese eel swimming behaviors were considered in this study: (a) diel vertical migration (DVM), (b) horizontal directed swimming toward settlement habitat, and (c) horizontal swimming depending on food availability in eddies (food attraction). Eel larvae undertake DVM behavior, i.e., they remain in the upper surface waters at night and dive to deeper waters during the day to avoid predators. Castonguay and McCleave^48^ observed *Anguilla* leptocephali (≥20 mm) in deep layers (125–275 m) during the day and in shallow layers (30–70 m) at night in the Sargasso Sea. Eel larvae observed in the STCC region have been predominantly leptocephali with body sizes larger than 20 mm^29^. Thus, simulated eel larvae were set to swim at a fixed depth of 50 m at night and to instantly move to and remain at the 200-m depth during the day. A random walk displacement was included to represent unresolved sub-grid turbulent flow and other non-resolved local processes^43^. The maximum random walk horizontal and vertical displacements in the present study were 600 m and 20 m, respectively. In the simulations, daytime was defined as sunrise (6 am local time) to sunset (6 pm local time) and nighttime was defined as sunset to sunrise.

Previously laboratory experiments demonstrated horizontal swimming speeds of approximately 0.036 ± 0.027 m s^−1^ for Japanese eel leptocephali^49^. In accordance with these results, horizontal swimming speeds between 0.01 and 0.06 m s^−1^ were used in our simulations. The mean swimming direction of eel larvae was westward (toward East Asia)^29,30^, and at each time step a random variation of up to 30° north or south of the mean direction was introduced to account for uncertainty in the swimming direction.

The food attraction scheme in our experiments represents the biological attraction of larvae to eddies due to the nature of eddies as food sources. Japanese eel larvae (leptocephali) feed on particulate organic matter such as marine snow^50^. Marine snow comprises detritus-like materials from all types of marine organisms. Therefore, detritus was used as a food source proxy in our simulations. Previous laboratory experiments have suggested that the feeding rate of herring larvae increased with food availability, resulting in altered swimming trajectories toward high food concentration areas^51^. However, as the food-attraction behavior of fish larvae is not well-documented, and the exact distance over which fish larvae can sense the presence of food remains unknown, the food-attraction function was programmed to activate once v-larvae encountered a food source in this work. It is also assumed that when v-larvae enter an eddy (the eddy margin is defined as sea surface height anomaly (SSHA) = + 0.02 m) and encounter food (a higher level of detritus than outside of the eddy), they attempt to change their swimming direction to remain in the eddy in order to continue feeding. In this circumstance, the v-larvae swim at the same speed toward the rich food zone, which was defined as the area within a 50-km radius from the maximum detritus position, assuming that v-larvae can sense the food concentration gradient. If the eddy interior contained less detritus than the exterior, the v-larvae kept their original westward heading.

It should be noted that JCOPE2 was not generated by a coupled bio-physical model and therefore the reanalysis data does not include biogeochemical variables. Because of this, SSHA was used as a proxy for food abundance in the realistic experiments by utilizing the negative relationship between SSHA and detritus (i.e., high detritus and low SSHA in cold (cyclonic) eddies and vice versa for warm (anti-cyclonic) eddies, as can be seen in the idealized experiments).

### Experimental design

Three sets of experiments were performed, including one idealized experiment and two sets of realistic experiments (Table 1). In the idealized experiment (Exp. 1), four cases were conducted to examine the physical and biological effects of mesoscale eddies on v-larvae dispersal. V-larvae were carried by ocean currents in warm or cold eddies, with or without food attraction. In each of the four cases, six different swimming speeds, 0.01, 0.02, 0.03, 0.04, 0.05, and 0.06 m s^−1^, were considered (Table 1). The idealized experiment without food attraction considered physical trapping only, and the experiment with food attraction considered both physical and biological effects. A total of 452 v-larvae were released inside the entire warm (or cold) eddy (defined as the area where the absolute SSHA was greater than 0.02 m) and tracked for 200 days.

**Table 1:** List of numerical experiments. “Phy” indicates physical trapping only, and “Phy + Food” represents combined effects of physical trapping and biological food attraction.

Exp. Name	Exp1				Exp2		Exp3	
Eddy	Idealized				Realistic, more cold eddies		Realistic, fewer cold eddies	
Eddy effects	Phy		Phy + food		Phy	Phy + food	Phy	Phy + food
Eddy type	cold	warm	cold	warm	mixed		mixed	
Swimming speed of v-larvae	0.01~0.06 m s^−1^				0.01~0.06 m s^−1^		0.01~0.06 m s^−1^	

**Figure 1:**
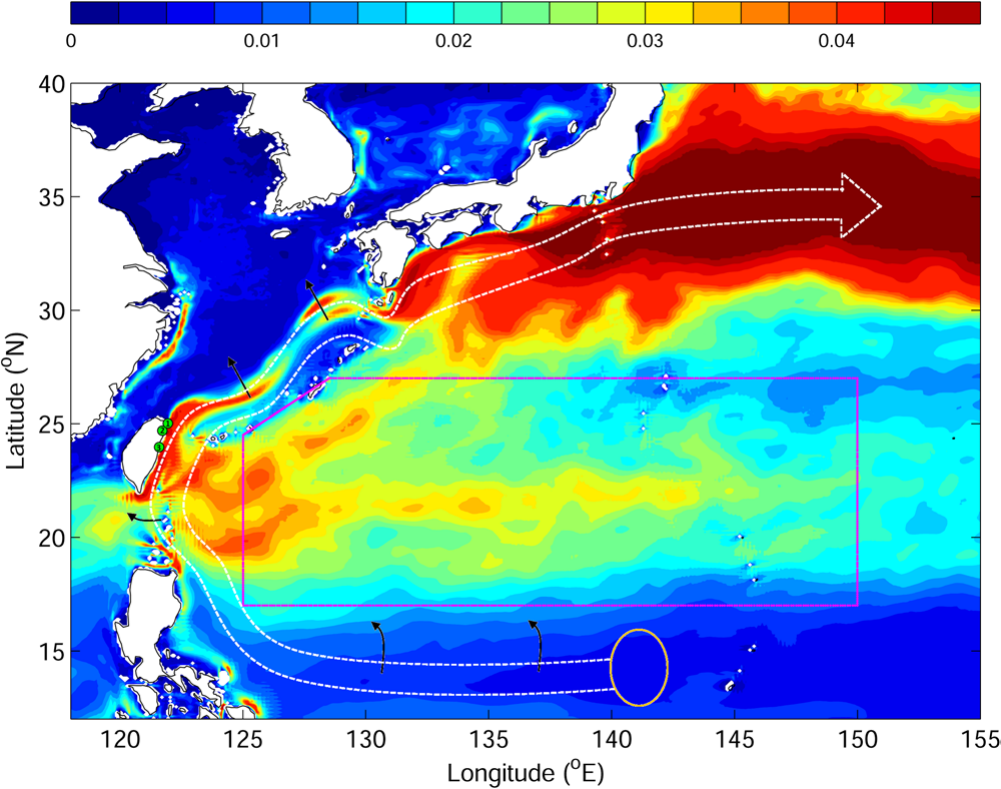
Eddy kinetic energy in the western North Pacific based on 20-year mean (1993-2012) JCOPE2 reanalysis. The magenta box is the local STCC eddy zone. Green circles mark the locations of glass eel collection. The gold oval, white dashed open arrow, and black arrows represent the Japanese eel spawning grounds, major ocean current path, and dispersion of Japanese eel larvae^3^, respectively. *Figure was created using MATLAB R2011b* (http://www.mathworks.com/).

In the realistic experiments, simulations were made for 2012 and 2010, when observations were available and the number of cold eddies was high (Exp. 2) and low (Exp. 3) respectively. Numerical simulations with and without food attraction were performed as in the idealized experiments. As in the idealized experiments, swimming speeds in the realistic experiments were varied from 0.01 to 0.06 ms^−1^. The v-larvae were released in the area within the STCC (west of 130°E, magenta box in Fig. 1) where eel larvae have been observed^29^. V-larvae were released in the STCC eddy zone rather than in the spawning ground to better assess the effect of eddies and to reduce any potential impacts of large-scale ocean currents such as the North Equatorial Current and the Kuroshio. A total of 5,342 v-larvae were released on September 1^st^ in each realistic experiment, which corresponds to the time of year when eel larvae have been observed in the STCC zone^29^, and were tracked until late February when the Taiwanese eel fishing season concludes.

### Observations

Japanese glass eels were caught by fishermen in Taiwan at several locations adjacent to the STCC eddy zone (green circles in Fig. 1), including Shungxi River Estuary (121.946°E and 25.020°N, station 1), Yilan River Estuary (121.835°E and 24.716°N, station 2), and Hualien River Estuary (121.611°E and 23.942°N, station 3). Following collection, the glass eels were stored in 95% alcohol for total length (TL) measurements. The annual mean TL, calculated using glass eel TLs acquired from November to the following February, was collected between 1999 and 2014 and was used as an indicator for eel larvae growth. All experimental procedures complied with the experimental animal ethics criteria approved by the Institutional Animal Care and Use Committee of National Taiwan University.

### Data availability

The datasets generated during and/or analyzed during the current study are available from the corresponding author upon reasonable request.

## Results

### Food source in eddies in the idealized experiments

**Figure 2:**
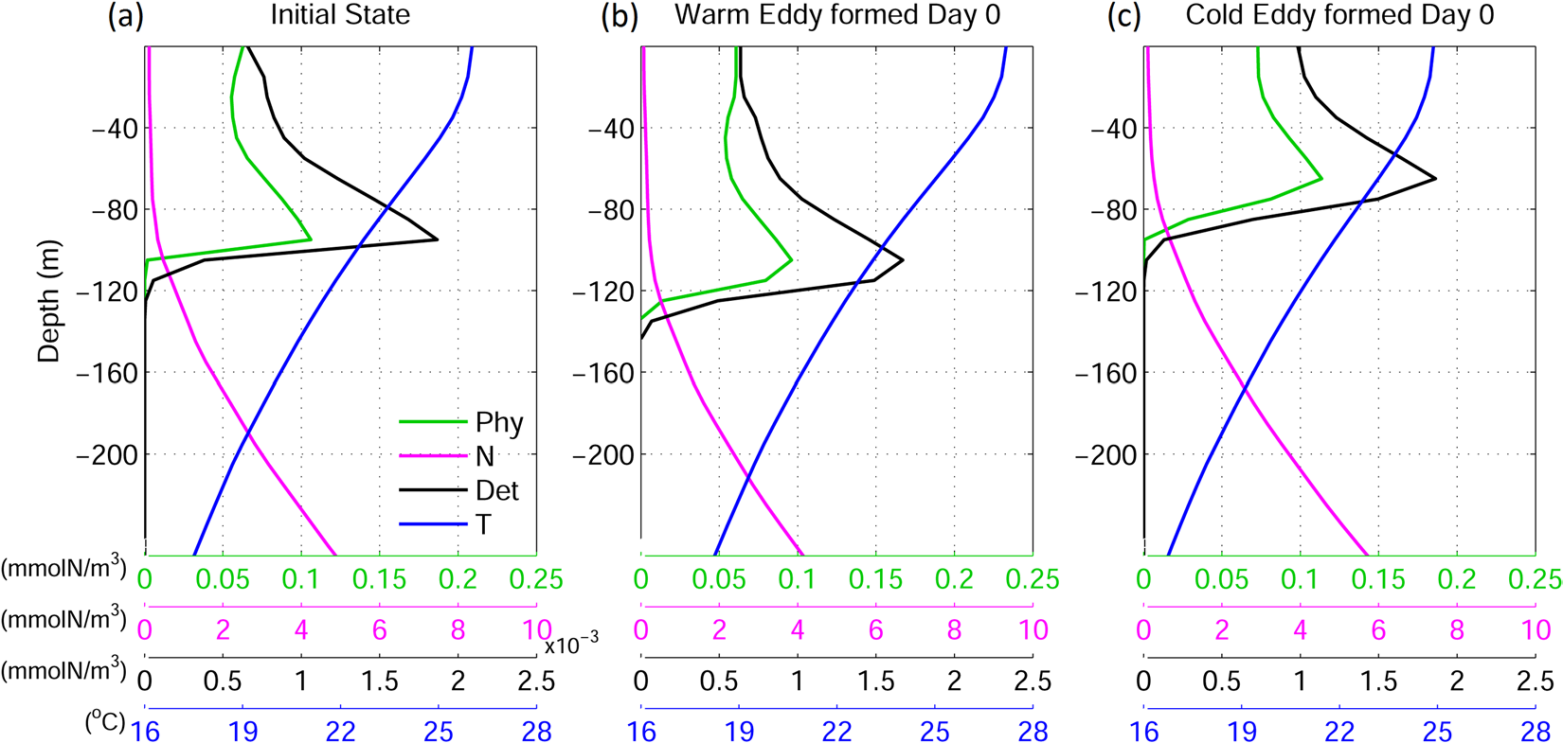
Vertical profiles of phytoplankton (green lines), nitrate (magenta lines), detritus (black lines, used as the food source index for eel larvae), and temperature (blue) in the idealized numerical experiments: (**a**) the initial state, (**b**) after the formation of the warm eddy, and (**c**) after the formation of the cold eddy. *Figure was created using MATLAB R2011b* (http://www.mathworks.com/).

**Figure 3:**
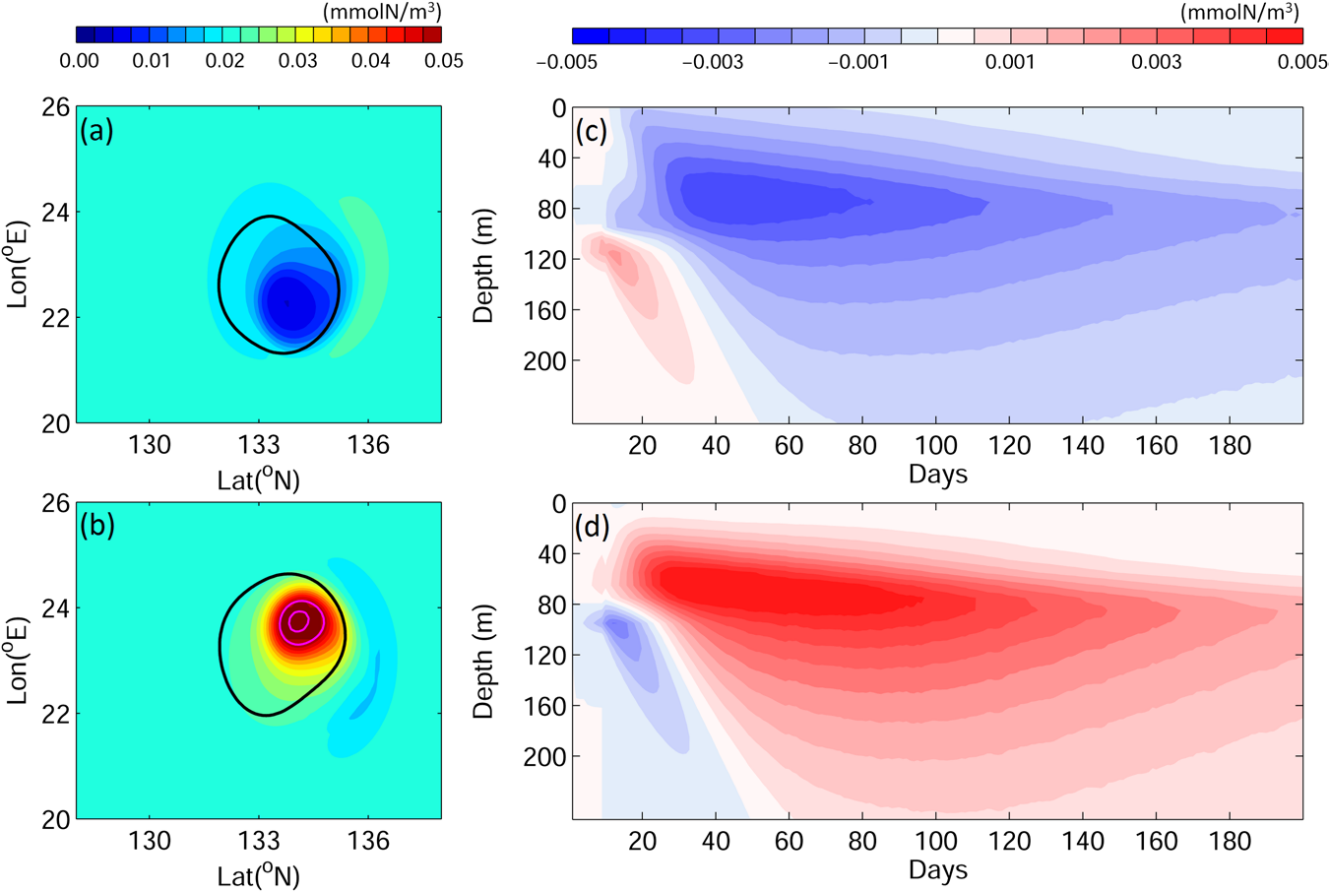
Distributions of vertically-integrated detritus in the top 200 m on day 70 in (**a**) the warm eddy and (**b**) the cold eddy; and time-depth distributions of detritus anomaly (calculated by subtracting the initial detritus distribution) in (**c**) the warm eddy and (**d**) the cold eddy in the idealized experiments. Black contour lines in (**a**) and (**b**) mark the eddy boundaries. *Figure was created using MATLAB R2011b* (http://www.mathworks.com/).

**Figure 4:**
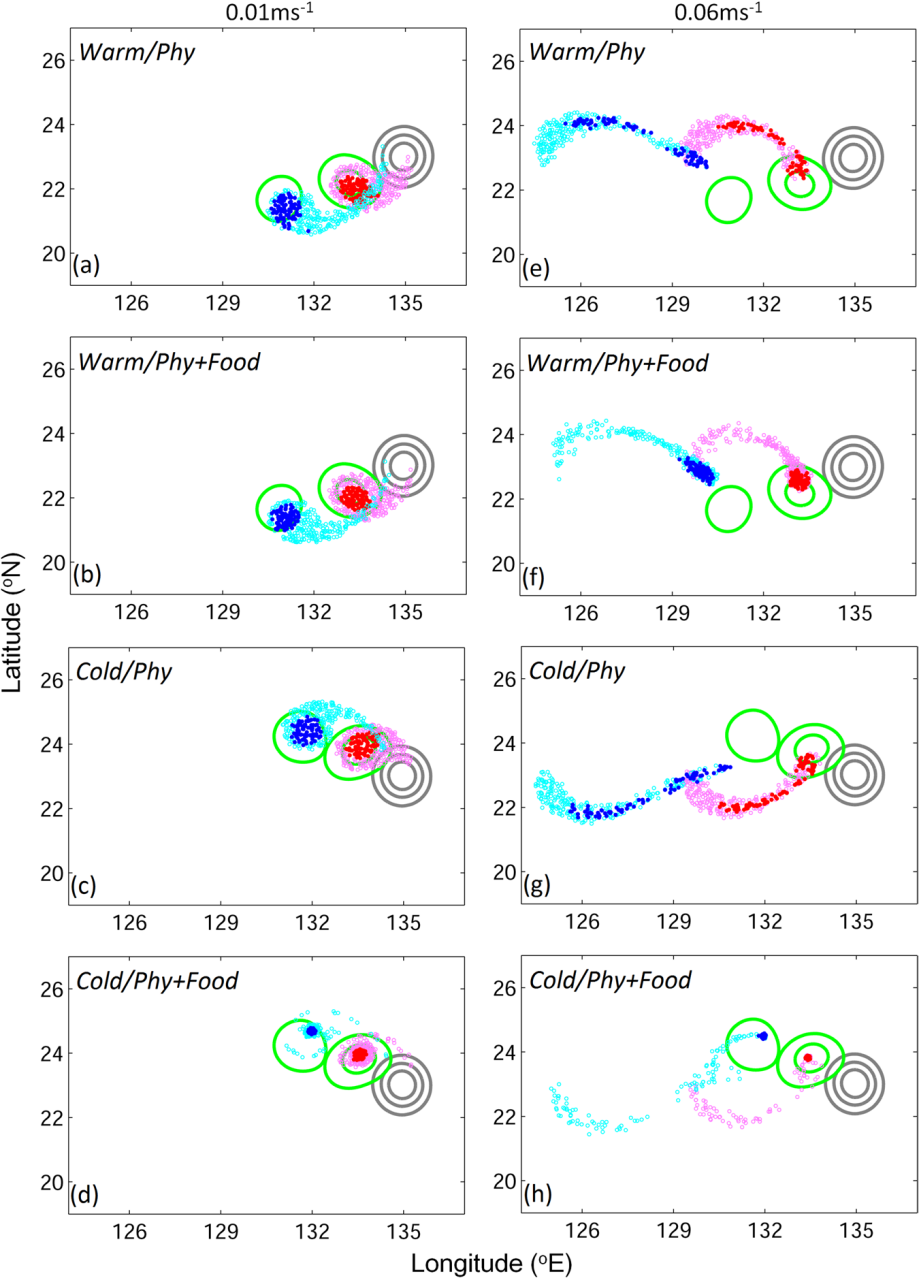
Distributions of v-larvae on day 100 (red, pink) and day 200 (blue, cyan) for swimming speeds of (left) 0.01 m s^−1^ and (right) 0.06 m s^−1^, in (**a,b,e,f**) the warm eddy, and (**c,d,g,h**) the cold eddy, with (**a,c,e,g**) physical trapping only and (**b,d,f,h**) physical trapping and food attraction, in the idealized experiments. The contours represent the SSHA, which mark the positions of eddies on day 0 (gray), and days 100 and 200 (green) respectively. Dark colors (red and blue) and light colors (pink and cyan) indicate v-larvae originating from the inner and outer parts of the eddy, respectively. *Figure was created using MATLAB R2011b* (http://www.mathworks.com/).

In the idealized experiments, the initial vertical concentration of nitrate was near-uniform in the top 80 m then increased with depth (magenta line in Fig. 2a). The initial phytoplankton and detritus concentrations reached their maxima at approximately 90 m (green and black lines in Fig. 2a). The initial water temperature was approximately 26 °C at the surface and decreased with depth to 19 °C at 200 m (blue line, Fig. 2a). During warm (Fig. 2b) and cold (Fig. 2c) eddy formation, isopycnals migrated downward and upward respectively. During this process, nutrients were redistributed in response to the isopycnal movement^52^; the downward movement in the warm eddy led to lower nitrate concentrations at a given depth (Fig. 2b), while the upward movement in the cold eddy transported nutrient-rich water to shallow layers (Fig. 2c). In response to the lower or higher nitrate concentrations, low and high phytoplankton concentrations (with respect to initial values) occurred in the near-surface waters (30–70 m) of warm and cold eddies respectively. Maximum phytoplankton concentrations were observed at 110 m and 75 m in the warm (green line in Fig. 2b) and cold (green line in Fig. 2c) eddies, respectively. The vertical profile of detritus, the food source proxy for eel larvae, underwent changes similar to those in phytoplankton (black lines, Fig. 2b,c). In the warm eddy, detritus concentration decreased in the upper 80 m and increased in the subsurface (80–140 m). Conversely, in the cold eddy, the detritus distribution was enhanced in the near-surface waters and reduced in deep water. The surface reduction (enhancement) and subsurface enhancement (reduction) of detritus in the warm (cold) eddy remained for ~40 days following eddy formation (Fig. 3c,d). Detritus evolved with nutrients, phytoplankton, and zooplankton after the eddy formation; after day 50, the detritus concentration in the entire water column of the warm eddy became lower than that of ambient water, with the minimum concentration occurring southeast of the eddy center (Fig. 3a). In contrast, detritus concentration increased throughout the cold eddy, with the maximum detritus concentration located northeast of the eddy center (Fig. 3b). The deviation of maximum and minimum detritus concentrations from the eddy center was due to a time lag between the eddy propagation and the response of biological processes. Therefore, the maximum and minimum detritus concentrations occurred at the eddy center positions of a few days prior (Figs 3 and 4). The low and high detritus in the warm and cold eddies, respectively, persisted for the entire duration of experiments (200 days).

### V-larvae distribution in eddies in the idealized experiments

Figure 4 presents distributions of v-larvae with two different swimming speeds under different trapping conditions in the warm and cold eddies. In the absence of food attraction, v-larvae retention in warm and cold eddies is mainly influenced by physical trapping in eddies and larvae’s swimming ability. In the case of the slow-swimming v-larvae (slower than the eddy propagation speed of 0.02–0.03 m s^−1^ in the idealized experiments), v-larvae that were initially placed in the eddy core were trapped in the eddies for all 200 days of the experiment, but some of the v-larvae that were initially placed in the outer part of an eddy escaped prior to day 100 and moved behind the eddy at their own swimming speeds (Fig. 4a,c). In the case of the fast-swimming v-larvae (faster than the eddy propagation speed), many larvae that were initially placed in the outer part of an eddy and some from the inner core escaped the eddies by day 100, and by day 200 all the fast-swimming v-larvae had escaped from the eddies (Fig. 4e,g). V-larvae distributions with the physical trapping effect only were similar between the warm and cold eddies, consistent with the previous studies^23^.

When food attraction was included in the models, both physical trapping and biological behavior contributed to v-larvae retention in eddies. In the warm eddy, v-larvae distribution was similar to the distribution without food attraction, particularly for slow-swimming v-larvae (Fig. 4a,b). Despite strong swimming abilities, fast-swimming v-larvae in the warm eddy were not retained until the end of the 200-day simulation due to low food availability. Nevertheless, the higher levels of detritus in the subsurface water during the early stages of the warm eddy (0–40 days) resulted in daytime v-larvae retention (Fig. 3). Therefore, the v-larvae stayed closer to the eddy than in the case with physical trapping only (Fig. 4e,f). In the cold eddy, v-larvae remained in the eddy for a long time due to the rich food source (Fig. 4d,h) and the v-larvae distributions were less dispersed than in the cases with physical trapping only (Fig. 4d,h vs. Fig. 4c,g). V-larvae in the cold eddy tended to move toward the food-rich zone, resulting in a concentrated distribution. Despite the plentiful food source in the cold eddy, several v-larvae were found outside the eddy. During the early stages of cold eddy experiments, lower detritus in the subsurface water was not conducive to v-larvae retention, and a portion of v-larvae left the eddy.

### Retention time in eddies in the idealized experiments

**Figure 5:**
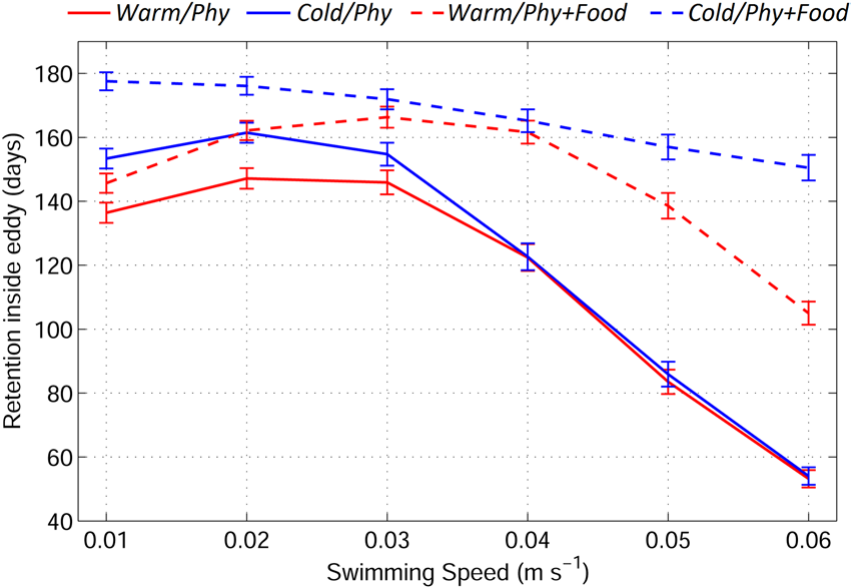
Relation between the v-larvae retention time and swimming speed in the warm (red) and cold (blue) eddy. The solid lines represent physical trapping only, and the dashed lines represent physical trapping with food attraction; the vertical bars represent standard deviation errors. *Figure was created using MATLAB R2011b* (http://www.mathworks.com/).

V-larvae influenced by physical trapping exhibited a similar retention time in warm and cold eddies when larval swimming speed is high. However, noticeable differences between the physical trapping abilities of warm and cold eddies when larval swimming speed is low were documented in a previous modeling study^23^. The relatively fast-moving warm eddy lost some of its nonlinearity (eddy rotation speed versus eddy propagation speed)^20^ due to a thicker upper layer, which decreased the eddy’s trapping ability and resulted in shorter retention times compared to the cold eddy (Fig. 5a). In cases with food attraction, the retention time was longer in the cold eddy, which had a richer food source than the warm eddy (Fig. 5a). V-larvae with food attraction generally stayed in eddies longer than those with physical trapping only. Nevertheless, the retention enhancement caused by food attraction was sensitive to the v-larvae swimming speeds. Slow-swimming v-larvae (0.01, 0.02, and 0.03 m s^−1^), whose swimming speeds are slower than or close to the eddy propagation speed (0.02~0.03 m s^−1^ in the idealized experiments), had approximately 7–16% longer retention times with food attraction. In comparison, the inclusion of food attraction increased the retention time of fast-swimming v-larvae by approximately 35% for a swimming speed of 0.04 m s^−1^ and about 178% for a swimming speed of 0.06 m s^−1^ in the cold eddy. Although v-larvae with stronger swimming abilities have a better chance of approaching food sources, the relationship between retention time and swimming speed is not linear, due to the eddies’ physical trapping effect. Therefore, the optimum swimming speed that allows v-larvae to remain in eddies is close or equal to the eddy propagation speed.

Both physical trapping and biological food attraction contributed to v-larvae retention in eddies. As mentioned above, physical trapping by the eddy depends on the ratio of v-larvae’s swimming speeds to eddy propagation speeds^23^; strong trapping occurs at low swimming speeds and results in longer eddy retention times. On the other hand, biological food attraction works in a way that is opposite from physical trapping. Fast-swimming v-larvae have a higher ability to approach food sources, which lengthens the retention time relative to physical trapping. Therefore, retention of slow-swimming v-larvae in the eddy is dominated by physical trapping, whereas biological food attraction prevails at high swimming speeds. An important question that arises is whether or not long eddy retention times correspond to increased feeding. Detritus concentrations in cold and warm eddies are different (Fig. 3). Therefore, long retention times in cold eddies can potentially result in increased feeding by the larvae. Conversely, long retention in warm eddies could lead to a decreased feeding. Nevertheless, v-larvae that undertook food attraction could obtain a larger amount of food than those that experienced physical trapping only, due to active directional swimming toward rich food zones (Fig. 4).

### Particle movements based on observations and realistic simulations

**Figure 6:**
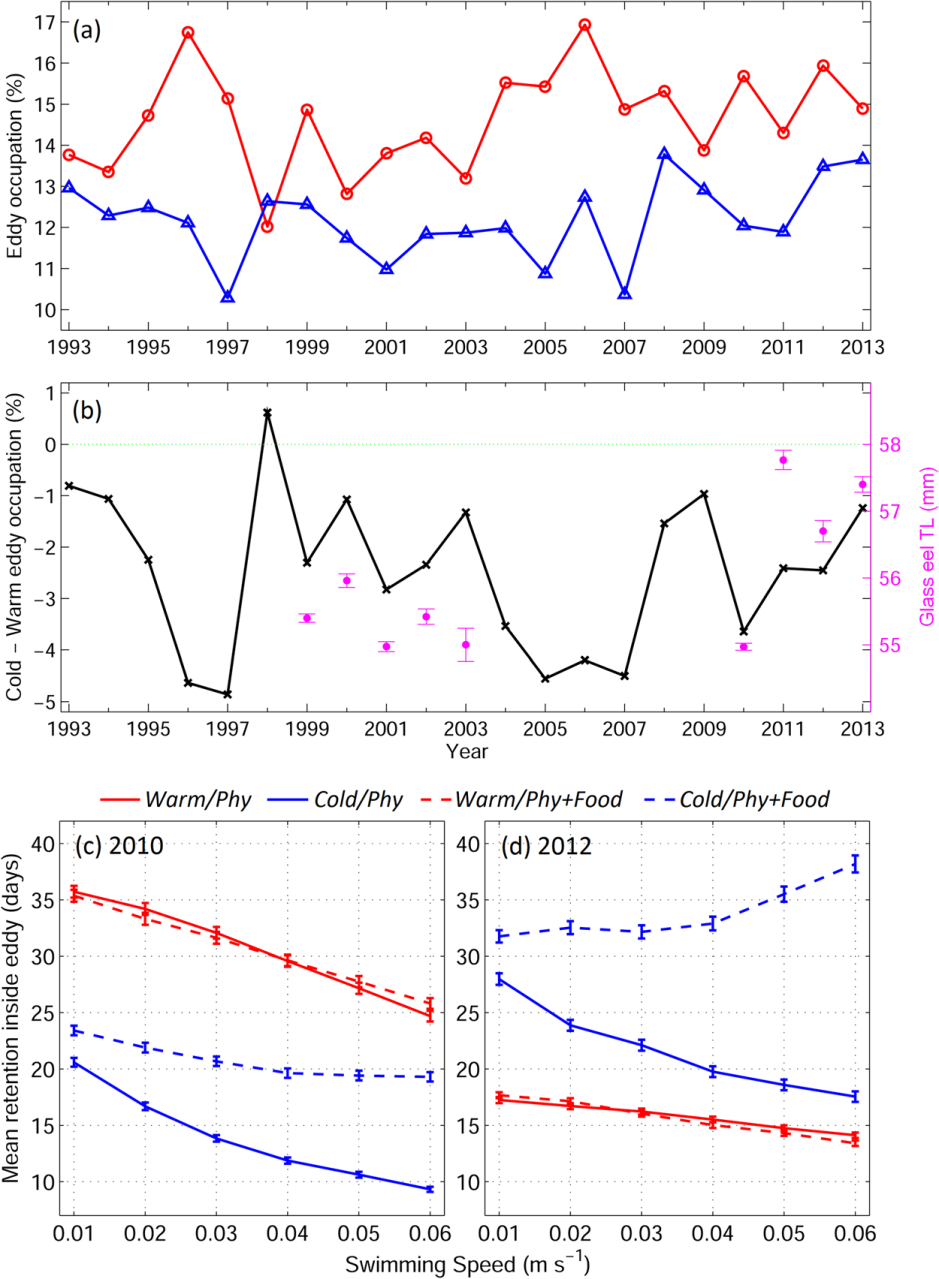
(**a)** Warm (red) and cold (blue) eddy occupation in the STCC region (west of 130^o^E) from 1993 to 2013 based on reanalysis data produced by JCOPE2. (**b**) Cold minus warm-eddy occupation (black line) and glass eel body length (magenta dots) observed in eastern Taiwan. V-larvae retention time in warm (red) and cold (blue) eddies with (dashed) and without (solid) food attraction for the year (**c**) 2010 and (**d**) 2012 versus swimming speeds from the realistic experiments. *Figure was created using MATLAB R2011b* (http://www.mathworks.com/).

Approximately 25–30% of the STCC area is covered by mesoscale eddies, with more warm than cold eddies (Fig. 6a,b). We simulated the dispersal trajectories of v-larvae in years with high (2012) and low (2010) numbers of cold eddies and calculated retention times using JCOPE2 reanalysis data from the realistic experiments. In 2012, v-larvae experienced a longer retention time in cold eddies than in 2010 (Fig. 6c,d), whereas the opposite relationship occurred in warm eddies. While warm eddies yielded very similar retention times with and without food attraction, v-larvae in cold eddies extended their stays when food attraction was included compared to v-larvae with physical trapping only. In 2012 (high number of cold eddies), the longer and shorter retention times compared to 2010 in cold and warm eddies, respectively, resulted in longer exposure times to food sources than in 2010 (low number of cold eddies) and may have led to fast larvae growth^18,19^. Furthermore, the glass eel body lengths measured in eastern Taiwan were longer in 2012 than in 2010, suggesting that mesoscale eddies may influence eel larvae dispersal and growth (Fig. 6b). Nevertheless, the relation between eddies and eel larvae dispersal were performed based on the two selected years, as eddies were not the only factor influencing the growth of eel larvae (see discussion), some years with high numbers of cold eddies might not correspond to the fast growth of glass eel.

## Discussion

In this study, we explored the effects of mesoscale eddies on fish larvae dispersal, including eddies’ ability to physically trap and transport the larvae and to act as a food source, which has direct applications to STCC mesoscale eddies and Japanese eel larvae in the western North Pacific Ocean. A three-dimensional (3D) particle-tracking method was used in which v-larvae were set to swim horizontally (with/without food attraction at various swimming speeds) and migrate vertically (DVM), in addition to being moved passively by ocean currents. Both idealized and realistic numerical experiments were conducted. In the idealized experiments, both physical trapping and biological food attraction contributed to v-larvae retention in eddies. Physical trapping dominated the retention of slow-swimming v-larvae (slower than eddy propagation), whereas biological food attraction was the dominant influence for fast-swimming v-larvae (faster than eddy propagation). Overall, v-larvae with biological food attraction tended to stay in mesoscale eddies longer than those with physical trapping only. As cold and warm eddies contain different amounts of food, similar retention times in warm and cold eddies led to different levels of food supply for fish larvae. Cold eddies provide a richer food supply than warm eddies and thus v-larvae with long retention times were able to obtain more food than their counterparts in warm eddies. In contrast, retention in warm eddies led to larvae encountering a smaller food supply compared to ambient water and cold eddies.

Early studies have illustrated that ocean productivity can influence larvae feeding success and survival, particularly during the early life stage^17,18^. Additionally, a previous study suggests that rich food supplies in mesoscale eddies lead to rapid growth of fish larvae and can enhance the survival and settlement of larval coral reef fish^19^. As there are multiple types of mesoscale eddies and warm or cold eddies can provide different amounts of food, trapping in warm eddies may result in reduced survival. On the other hand, fish larvae retention in mesoscale eddies may attract predators^53^, which introduces uncertainty in survival rates.

This study considered the physical and biological effects of eddies on fish larvae. Behavior such as swimming speed, DVM, and feeding^54–56^ may vary between adult and larval eels, and the relative importance of physical trapping versus biological food attraction could differ by life stage and/or by species. For example, the adult Japanese eel can swim faster and dive deeper than the larvae^48,57^. These abilities allow adult eel to avoid physical trapping^23^ and escape the eddy (the vertical extent of STCC eddies is 400–600 m). Thus, fast swimming dampens the importance of physical trapping and biological food attraction may become the controlling factor.

Our realistic experiments demonstrated that during the year 2012, which had a high number of cold eddies, v-larvae have longer retention times in cold eddies than in warm eddies regardless of food attraction. This led to a greater exploitation of the food supply in our simulations and may have resulted in the observed larger body lengths of glass eels than during the year 2010, which had fewer cold eddies. Although the food attraction simulations assumed that eel larvae can sense and approach food, the reality of this behavior in nature is unknown. V-larvae with food attraction can remain in cold eddies longer and therefore obtain more food than the case with physical trapping only. Therefore, more knowledge of fish larvae’s attraction to food is necessary in order to better understand and simulate the biological role of mesoscale eddies in fish larvae dispersal.

Although the two selected years demonstrated a potential relation between eddies and glass eel body length, exceptions were noticed in other years. The realistic ocean conditions included complex dynamic processes and 35–55% of the v-larvae did not encounter any eddies during the 180-day simulations. This suggests that the rich food sources in cold eddies are not necessarily used by all larvae. Moreover, v-larvae were influenced by background ocean currents in locations without eddies, and v-larvae that reached the east coast of Taiwan may have originated in southerly areas (e.g., the North Equatorial Current region) and may not have entered the STCC eddy zone. Besides, this study did not consider other biological factors such as settlement processes, which can also influence lengths of glass eel. A lack of observational fish larvae data from the open ocean makes it difficult to verify our hypothesis at present. Therefore, direct tracking of fish larvae in mesoscale eddies is necessary to validate our results and to better understand the impact of eddies on fish larvae migration.
